# Protective Effects of Amarogentin against Carbon Tetrachloride-Induced Liver Fibrosis in Mice

**DOI:** 10.3390/molecules22050754

**Published:** 2017-05-06

**Authors:** Ya Zhang, Hang Zhao, Hua Li, Wei Cao, Fang Wang, Tian Zhang, Si-Wang Wang

**Affiliations:** 1 Department of Natural Medicine, School of Pharmacy, Fourth Military Medical University, 169 West Changle Road, Xi’an 710032, China; lmlx2428@163.com (Y.Z.); zhaohang169@163.com (H.Z.); lihuasmile@aliyun.com (H.L.); caowei@fmmu.edu.cn (W.C.); wangfang3463704@163.com (F.W.); 2 Xi’an Day Natural Inc., F501 Gazelle Valley, Pioneering R&D Park, 69 Jinye Road, Xi’an 710077, China; tian@daynatural.com

**Keywords:** amarogentin, carbon tetrachloride, liver fibrosis, α-smooth muscle actin, mitogen-activated protein kinase

## Abstract

Amarogentin, a secoiridoid glycoside that is mainly extracted from *Swertia* and *Gentiana* roots, has been suggested to exhibit many biological effects, including anti-oxidative, anti-tumour, and anti-diabetic activities. The present study was designed to evaluate the protective effects of amarogentin on carbon tetrachloride-induced liver fibrosis in vivo and the underlying mechanism. Fibrosis was induced by subcutaneous injections of 6 mL/kg of 20% carbon tetrachloride (dissolved in olive oil) twice per week for seven weeks. Mice were orally treated with 25, 50, and 100 mg/kg amarogentin and with colchicine as a positive control. Biochemical assays and histopathological investigations showed that amarogentin delayed the formation of liver fibrosis; decreased alanine aminotransferase, aspartate aminotransferase, malondialdehyde and hydroxyproline levels; and increased albumin, cyclic guanosine monophosphate, glutathione peroxidase, and superoxide dismutase levels. Moreover, amarogentin exhibited downregulation of α-smooth muscle actin and transforming growth factor-β_1_ levels in immunohistochemical and Western blot analyses. The levels of phosphorylated extracellular regulated protein kinases, c-Jun N-terminal kinase, and p38 were also significantly reduced in all amarogentin-treated groups in a dose-dependent manner. These findings demonstrated that amarogentin exerted significant hepatoprotective effects against carbon tetrachloride-induced liver fibrosis in mice and suggested that the effect of amarogentin against liver fibrosis may be by anti-oxidative properties and suppressing the mitogen-activated protein kinase signalling pathway.

## 1. Introduction

Liver fibrosis results from a continuous wound-healing response of the liver to repeated injury, such as chronic hepatitis B or C infections, chemical intoxication, alcohol abuse, metabolic syndrome, or autoimmune diseases [1]. Chronic liver injury disrupts the balance between the production and degradation of extracellular matrix (ECM) and subsequently leads to liver fibrosis and cirrhosis. Without effective therapy, liver injury may ultimately develop into hepatic failure or hepatocellular carcinoma (HCC) [2]. Liver fibrosis and cirrhosis remain major causes of morbidity and mortality worldwide and have increasing economic and social impacts [3], but efficient anti-fibrotic drugs with few side effects have not been discovered. The current treatment for hepatic fibrosis is limited to withdrawal of the noxious agents and orthotropic liver transplantation in the late stages. Thus, the search for new therapeutic drugs continues. A better understanding of the molecular mechanism of liver fibrosis would facilitate the development of preventive and therapeutic approaches for liver fibrosis and HCC.

Liver injury stimulates a variety of cytokines and growth factors that can activate hepatic stellate cells (HSCs) to produce α-smooth muscle actin (α-SMA) and ECM, resulting in liver fibrosis. Transforming growth factor-β_1_ (TGF-β_1_) has been considered as the most important profibrogenic cytokine, which contributes to the development of liver fibrosis through modulation of both synthesis and degradation of ECM proteins [4]. Additionally, an important cell signalling pathway, the mitogen-activated protein kinase (MAPK) pathway, and its subsidiary extracellular regulated protein kinases (ERK), c-Jun N-terminal kinase (JNK), and p38 pathways, are involved in the aggravation of hepatic fibrosis; thus, these pathways may be targets for treating hepatic fibrosis [5]. Carbon tetrachloride (CCl_4_), a well-known hepatotoxin, is commonly used in laboratory animals to induce liver injuries, including fibrosis. The molecular basis is the metabolic conversion of CCl_4_ to highly-reactive CCl_3_· radicals by cytochrome P450 enzymes [6]. These radicals initiate radical-mediated lipid peroxidation prior to damaging the liver cell membrane and inducing acute liver injury [7]. It has been reported that products from natural and medicinal plants have a high capacity for scavenging CCl_4_-induced free radicals [8]. Numerous purified compounds and formulae have been shown to exert anti-fibrotic properties [9,10] and have been used in China for thousands of years to treat liver disease [11,12].

**Figure 1:**
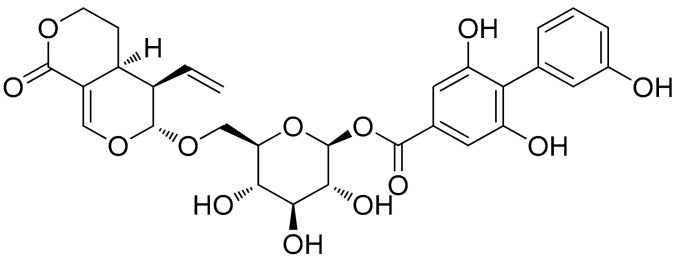
Chemical structure of amarogentin (AG, chemical name: (4a*S*,5*R*,6*S*)-5-ethenyl-4,4a,5,6-tetrahydro-6-[[2-*O*-[(3,3′,5-trihydroxy[1,1′-biphenyl]-2-yl)carbonyl]-β-d-glucopyranosyl]oxy]-1*H*,3*H*-pyrano[3,4-*c*]pyran-1-one.

Amarogentin (AG, Figure 1), a secoiridoid glycoside that is mainly extracted from *Swertia* and *Gentiana* roots [13,14], exhibits many biological effects, including anti-oxidative, anti-tumour, and anti-diabetic activities. As a folk medicine, *Swertia mileensis* T. N. Ho and W. L. Shih (*Gentianaceae*) has been used to treat viral hepatitis in China for centuries [15], and *Swertia chirayita* (Roxb. ex Fleming) H. Karst. exerts hepatoprotective effects against CCl_4_- and paracetamol-induced damage in rats [16]. As an active component of these two plants, recent studies have demonstrated that AG can inhibit liver carcinogenesis by regulating the activity of the G_1_/S cell cycle checkpoint kinase and inducing apoptosis at mild dysplastic stage [17]. These studies all indicate the hepatoprotective effects of AG.

However, the mechanism of the hepatoprotective effects of AG remains unclear. In the present study, the protective effects of AG on CCl_4_-induced liver fibrosis were evaluated. The possible anti-fibrotic mechanisms by which AG regulated oxidative stress and the MAPK pathways were also investigated.

## 2. Results 

### 2.1. Effect of AG on HSC Proliferation and Apoptosis

HSC proliferation was measured by Cell Counting Kit-8 (CCK-8) assay, and the protein expression of B-cell lymphoma-2 (Bcl-2) and Bcl-2 Associated X protein (Bax) were determined by Western blot to evaluate the apoptosis in activated HSC. The results showed that there is no significant inhibitory effect of AG on HSC proliferation. However, AG can significantly, dose-dependently, suppress TGF-β_1_-induced HSC proliferation and accelerate the apoptosis of HSC by the increasing level of Bax protein and the decreasing level of Bcl-2 as compared to the TGF-β_1_ group (*p* < 0.05) (Supplementary Figure S1).

### 2.2. Effects of AG on the Relative Liver Weights and Liver Function in CCl_4_-Treated Mice

As shown in Table 1, the relative liver weights of the CCl_4_ group were significantly higher than the control group (*p* < 0.05), but no statistically significant difference was observed between the AG and colchicine groups (*p* > 0.05). Compared with the CCl_4_ group, the 100 mg/kg AG group showed a significant decrease in relative liver weight (*p* < 0.05). This observation is in agreement with previous results [18]. The alanine aminotransferase (ALT) and aspartate aminotransferase (AST) levels were significantly higher, and the albumin (Alb) level was significantly lower (*p* < 0.05) in the CCl_4_ group than in the control group. The AG (25, 50, 100 mg/kg) and colchicine treatments significantly decreased ALT and AST levels (*p* < 0.05) and increased Alb levels, while the 100 mg/kg AG group made significantly different levels (*p* < 0.05) than the CCl_4_ group. Cyclic guanosine monophosphate (cGMP) levels were significantly higher in the AG groups than in the CCl_4_ group (*p* < 0.05). Among the experiment, one mouse died in the CCl_4_ group and AG (25 mg/kg) group, respectively (Supplementary Table S1). In the acute toxicity study, oral administration of AG at a dose of 1000 mg/kg did not produce any signs of toxicity and no animals died for up to 14 days.

### 2.3. Effects of AG on Liver Lipid Peroxidation and Antioxidant Capacity

**Figure 2:**
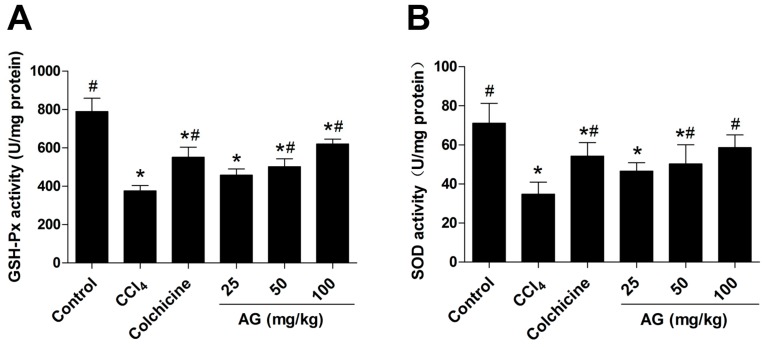

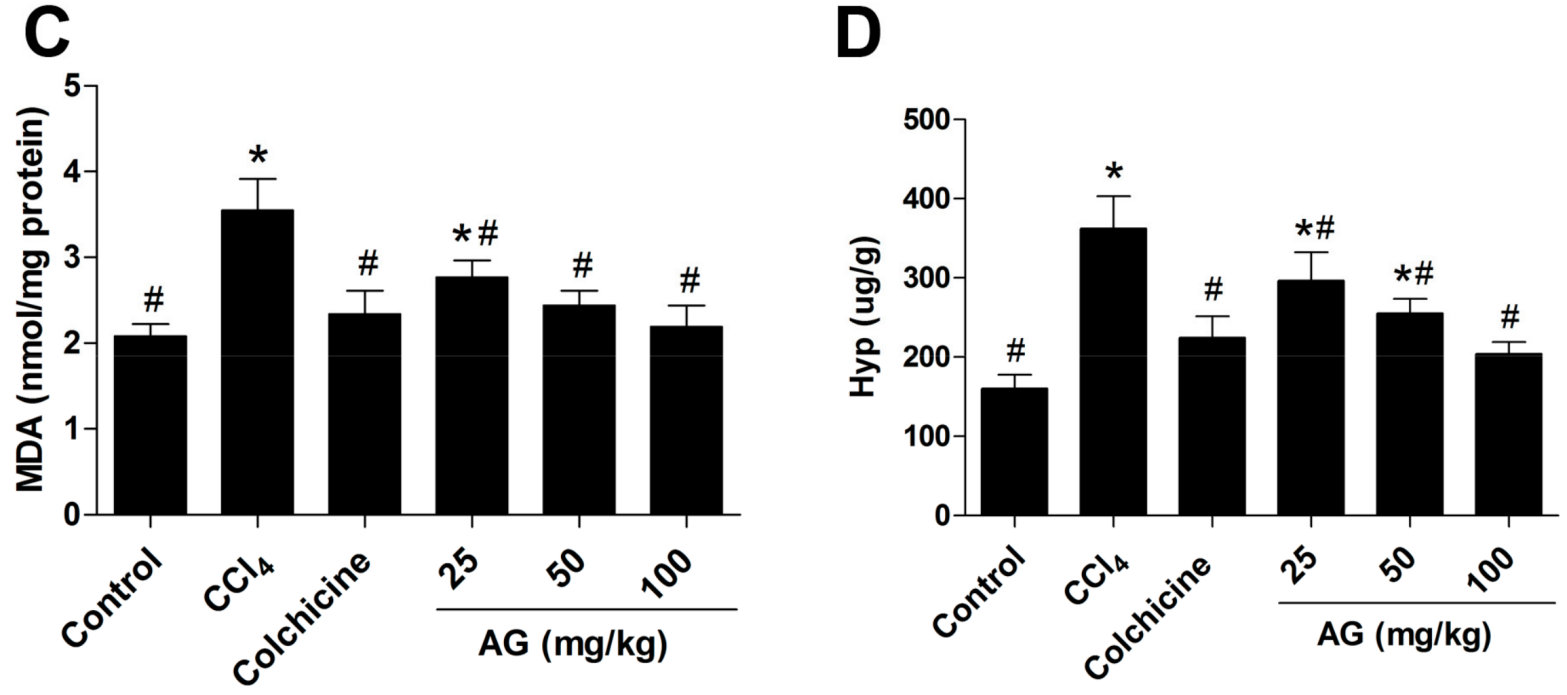
(**A**) Glutathione peroxidase (GSH-Px), (**B**) superoxide dismutase (SOD), (**C**) malondialdehyde (MDA), and (**D**) hydroxyproline (Hyp) levels in liver tissues. Values are expressed as the means ± SD (*n* = 7–8) and were analysed using one-way ANOVA with Tukey’s multiple comparisons test. * *p* < 0.05 versus the control group, ^#^ *p* < 0.05 versus the CCl_4_ group.

The glutathione peroxidase (GSH-Px), superoxide dismutase (SOD) and malondialdehyde (MDA) levels in liver tissues were measured to determine the antioxidant effects of AG, and the results are shown in Figure 2A–C, respectively. Significantly higher (*p* < 0.05) GSH-Px (Figure 2A) and SOD (Figure 2B) levels were observed in the AG (50, 100 mg/kg) and colchicine groups than in the CCl_4_ group. Additionally, MDA (Figure 2C) levels in the AG (25, 50, 100 mg/kg) and colchicine groups were significantly lower (*p* < 0.05) than in the CCl_4_ group, as expected. The hydroxyproline (Hyp) (Figure 2D) content was markedly higher in the CCl_4_ group than in the control group (*p* < 0.05). Hyp levels were significantly lower in the AG (25, 50, 100 mg/kg) and colchicine groups than in the CCl_4_ group (*p* < 0.05).

### 2.4. Effects of AG on Histological Changes

**Figure 3:**
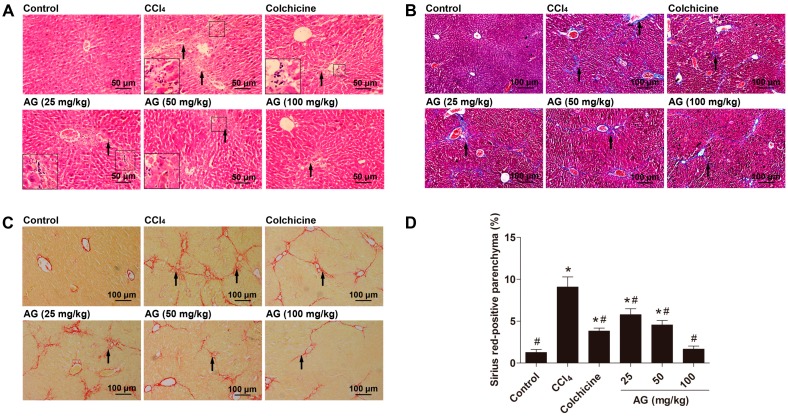
Effects of AG on CCl_4_-induced liver fibrosis in mice. (**A**) Liver fibrosis assessed by haematoxylin and eosin staining (200×); (**B**) liver fibrosis assessed by Masson’s Trichrome staining (100×); (**C**) liver fibrosis assessed by Sirius Red staining (100×); and (**D**) quantitative analysis of Sirius red staining using ImageJ (Bio-Rad, Hercules, CA, USA). The arrows indicate the areas of CCl_4_-induced collagen deposition. Values are expressed as the means ± SD (*n* = 7–8) and were analysed using one-way ANOVA with Tukey’s multiple comparisons test. * *p* < 0.05 versus the control group, ^#^ *p* < 0.05 versus the CCl_4_ group.

Histopathological evaluations are regarded as the best method for assessing the severity of hepatic fibrosis. We evaluated liver sections with haematoxylin and eosin, Masson’s Trichrome and Sirius Red staining to determine the extent of hepatic fibrosis. According to haematoxylin and eosin staining (Figure 3A), the liver tissues of the control group showed normal lobular architecture with central veins and radiating hepatic cords, with no regenerating collagen fibres. In the CCl_4_ group, extensive degeneration, a dense inflammatory infiltrate, and the disappearance of the normal structure of the lobes were observed. Liver tissues in the AG and colchicine group showed less necrosis of hepatic cells and less collagen deposition than the model group. In addition, after Masson’s Trichrome staining (Figure 3B), collagen fibres in the model group were largely enhanced compared with the normal group, but were markedly decreased in the AG and colchicine groups. The extent of liver fibrosis was also documented by Sirus Red staining (Figure 3C). A marked increase in Sirius Red staining (red staining) was observed in the livers of the CCl_4_ group compared with the control group. By contrast, the increase in Sirius Red staining was markedly reduced by the AG and colchicine treatments (Figure 3D).

### 2.5. Effects of AG on the Expression of α-SMA and TGF-β_1_

**Figure 4:**
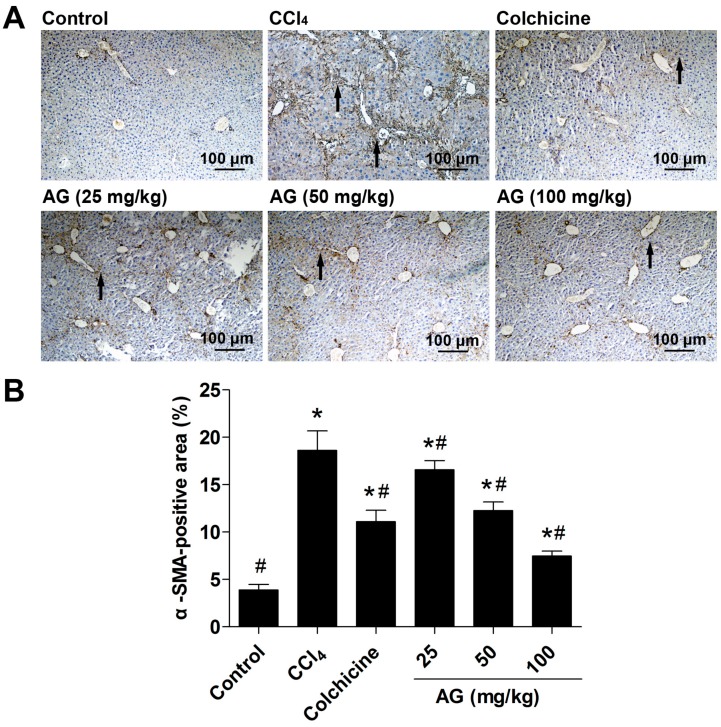
Effect of AG on α-smooth muscle actin (α-SMA) expression in CCl_4_-treated mice. (**A**) Representative immunohistochemical staining of α-SMA expression in liver tissue; (**B**) quantitative analysis of α-SMA staining using ImageJ (Bio-Rad, Hercules, CA, USA). The arrows indicate the activated hepatic stellate cells in tissue. Values are expressed as the means ± SD (*n* = 7–8) and were analysed using one-way ANOVA with Tukey’s multiple comparisons test. * *p* < 0.05 versus the control group, ^#^ *p* < 0.05 versus the CCl_4_ group.

**Figure 5:**
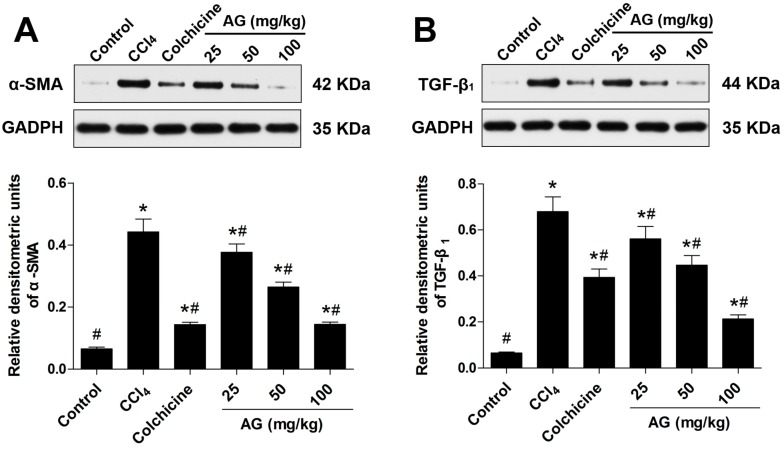
Effects of AG on the CCl_4_-induced hepatic accumulation of α-SMA and transforming growth factor-β_1_ (TGF-β_1_) proteins in mice. Band intensities were quantified by densitometry. (**A**) Immunoblot and densitometry analysis of α-SMA expression; and (**B**) immunoblot and densitometry analysis of TGF-β_1_ expression. Values are expressed as the means ± SD (values of three independent experiments) and were analysed using one-way ANOVA with Tukey’s multiple comparisons test. * *p* < 0.05 versus the control group, ^#^ *p* < 0.05 versus the CCl_4_ group. GAPDH: glyceraldehyde-3-phosphate dehydrogenase.

Hepatic stellate cells are regarded as the most relevant cell for the development of liver fibrosis, and their activation is the key step in the process of liver fibrogenesis [19]. α-SMA, the marker of activated HSCs following liver injury, were used to evaluate the degree of HSC activation by immunohistochemical staining and Western blots. Chronic CCl_4_ treatment significantly increased the accumulation of α-SMA compared with the control group (*p* < 0.05). Compared with the CCl_4_ group, the AG treatment significantly decreased α-SMA (Figure 4 and Figure 5A) (*p* < 0.05). TGF-β_1_ has been recognized as a key role in hepatic fibrosis [4]. As shown in Figure 5B, the expression of TGF-β_1_ was increased in the CCl_4_-treated group compared with the control group (*p* < 0.05). The levels of TGF-β_1_ decreased compared with those of the CCl_4_-treated group when AG was co-administered (*p* < 0.05).

### 2.6. Effects of AG on MAPK Phosphorylation

**Figure 6:**
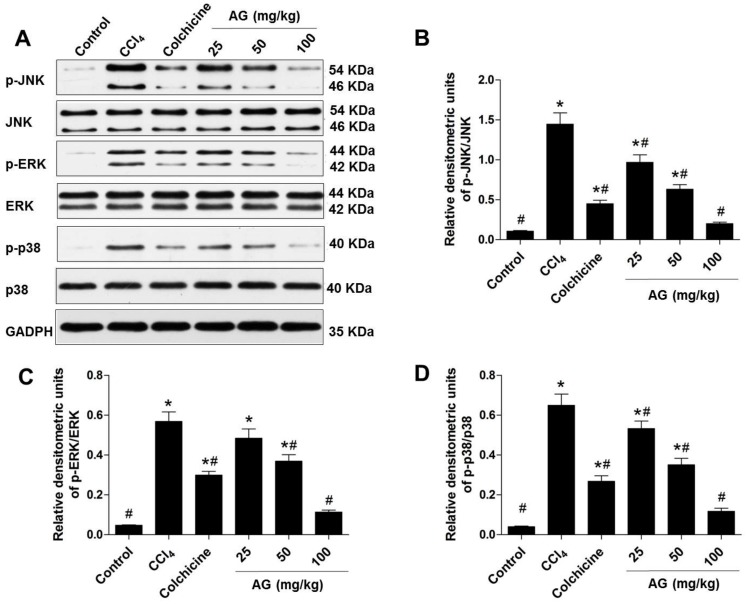
Effect of AG on the mitogen-activated protein kinase (MAPK) pathway in the CCl_4_ model. Band intensities were quantified by densitometry. (**A**) Immunoblots of the p-c-Jun N-terminal kinase, (p-JNK), p-extracellular regulated protein kinases (p-ERK), and p-p38 protein expression levels in liver tissues; (**B**) densitometry analysis of p-JNK/JNK; (**C**) densitometry analysis of p-ERK/ERK; and (**D**) densitometry analysis of p-p38/p38. Values are expressed as the means ± SD (values of three independent experiments) and were analysed using one-way ANOVA with Tukey’s multiple comparisons test. * *p* < 0.05 versus the control group, ^#^ *p* < 0.05 versus the CCl_4_ group.

The MAPK signalling pathways play a critical role in CCl_4_-induced liver fibrosis. The expression and phosphorylation of JNK, ERK, and p38 were examined by Western blotting to further investigate the role of the MAPK pathway in regulating hepatic fibrosis. As shown in Figure 6A–D, CCl_4_-induced JNK, ERK, and p38 phosphorylation was markedly suppressed by AG in a dose-dependent manner (*p* < 0.05). The results of Western blot were consistent with the results of the histopathology, biochemical, and immunohistochemical analyses in a dose-dependent manner.

## 3. Discussion

Hepatic fibrosis is a condition in which excessive cell proliferation and abnormal deposition of ECM in hepatic tissues cause structural and functional pathological changes in the liver. The mouse model of CCl_4_ resembles all important properties of human liver fibrosis, including inflammation, regeneration, fibre formation and, potentially, fibrosis regression. Most studies still rely on the CCl_4_-model to induce toxic liver fibrosis in mice due to numerous previous publications for comparison, excellent reproducibility of the model, and the moderate burden to the animals [20]. This model was, therefore, chosen as the representative model in this study. Currently, the anti-fibrotic activity of AG in CCl_4_-induced animal models has not been reported. The present study is the first to show that AG has efficacious inhibitory properties against CCl_4_-induced hepatic fibrosis in C57BL/6 mice. We firstly investigate that the possible anti-fibrotic mechanisms of AG may be by anti-oxidative properties, decreasing HSC activation by HSC apoptosis and suppressing the MAPK signalling pathway. Colchicine is a microtubule disrupting agent; it binds to tubulin, inhibits polymerization of microtubules, interferes with the transcellular movement of collagen, reduces the activity of hepatic collagen-processing enzymes, and stimulates the production of collagenase. Due to these composite effects, colchicine has been used for the treatment of liver disease characterized by prominent tissue inflammation or fibrosis [21,22]. Although colchicine exerts anti-inflammatory, anti-fibrotic, and immunomodulatory effects, it is not suitable to treat patients because important adverse events have been described [23]. In the present study, colchicine was used as a suitable positive control.

Alanine aminotransferase and aspartate aminotransferase are well-known indicators of liver injury. These two enzymes are released into the blood when the liver is injured [24]. AG supplementation reduced the ALT and AST levels and increased Alb activity compared with the CCl_4_ group. These results suggested that AG exhibits protective effects against CCl_4_-induced liver fibrosis by improving the serum parameters of liver function. The development of CCl_4_-induced liver fibrosis is usually associated with oxidative stress and lipid peroxidation. Since the oxidant/antioxidant imbalance is thought to be a key step in the development of fibrosis, oxidative stress has been carefully investigated in this study [25,26]. Hepatic MDA, SOD, and GSH-Px are indicators of oxidative stress and play important roles in the development of hepatic fibrosis. As a major component of the collagen protein, Hyp is produced through the hydroxylation of the amino acid proline [27]. Many studies have demonstrated that increased Hyp levels promote liver fibrosis. Here, we demonstrated that AG decreased MDA and Hyp levels and increased SOD and GSH-Px levels. It was reported that AG exhibits potent antioxidant activities against various diseases due to the unique structure of secoiridoid glycoside, including the catechol moiety and the completely conjugated system [28,29]. Nitric oxide (NO) has scavenged reactive oxygen species capabilities. In the present study, measurements of cGMP, a marker of NO bioavailability, were performed in mice liver homogenates. We found that AG treatment increased hepatic cGMP levels (reflecting hepatic NO bioavailability). This phenomenon is in agreement with previous results [30]. These results suggested that AG exerted an antioxidant effect in this study due to increasing the oxygen radical-absorbing capacity of the cells and enhancing their antioxidant activity. Liver histology is the clinical gold standard for evaluating the degree of hepatic injury in vivo. We have observed that AG treatment significantly reduced the CCl_4_-induced pathological changes and accumulation of collagen fibres. Thus, AG has the potential to attenuate CCl_4_-induced liver injury. Most importantly, toxicity studies, including blood pathology, histological staining of tissues, and specific enzyme levels, indicated that AG supplementation did not have negative effects on the mouse liver [31]. The acute toxicity study showed that AG was nontoxic in treated mice with a maximum dose up to 1000 mg/kg body weight.

Hepatic stellate cells are the primary cell type in the liver responsible for excess collagen synthesis during liver fibrosis [11]. In the normal liver, quiescent HSCs are located in the perisinusoidal space of Disse and serve as the major storage site of lipid-soluble vitamin A. Upon injury, quiescent HSCs transform into the proliferative and α-SMA-expressing myofibroblast phenotype that synthesizes ECM proteins [32,33]. α-SMA has been shown to be a fairly reliable marker of HSC activation in both experimental and clinical settings [6]. Our data indicate that CCl_4_ treatment increased α-SMA and initiated hepatic fibrogenesis, while AG decreased α-SMA expression. Based on a previous study, the liver is protected from further damage during liver fibrosis via inactivation of HSCs [34]. We predict that AG decreases the activation of HSCs, resulting in the reduction in α-SMA expression. As one of the main regulators of α-SMA is TGF-β_1_ [4,35], we next examined if different doses of AG are able to inhibit TGF-β_1_ expression in mice with CCl_4_-induced liver fibrosis. According to the results shown, CCl_4_ treatment increased the expression levels of TGF-β_1_, whereas AG significantly suppressed its expression. This effect may provide an advantage to liver to escape the increasing fibrosis growth signals of TGF-β_1_ and may be linked to critical steps in the progression of wound repair in the liver. Several studies have provided ample evidence that TGF-β_1_ is a strong stimulant of HSC proliferation [36]. Therefore, we used TGF-β_1_ to stimulate HSC. Our results indicate that AG significantly inhibits TGF-β_1_-induced HSC proliferation in cultured HSCs, suggesting that AG exhibits potential therapeutic benefits in ameliorating hepatic fibrosis. Cell apoptosis involves at least two major pathways. One is the death receptor pathway. The other is the mitochondrial pathway. Bcl-2 family members play an important role in regulating mitochondria-mediated apoptosis [37]. The release of pro-apoptotic factors from mitochondria is controlled by Bcl-2 and Bax [38]. Bcl-2 is an integral membrane protein with anti-apoptotic activity and increases the resistance of cells to apoptosis [39]. Bax is one of the pro-apoptotic members of the Bcl-2 family. Our result showed that AG can significantly accelerate TGF-β_1_-induced apoptosis of HSC, possibly by upregulating Bax expression and downregulating Bcl-2 expression, thus providing a pro-apoptosis strategy against liver fibrosis.

The MAPK signalling pathways play an important role in CCl_4_-induced liver fibrosis. When MAPKs are activated, they can translocate into the nucleus and activate several transcription factors, leading to various cellular responses such as proliferation, differentiation, and regulation of specific metabolic pathways [5,40]; thus, AG and CCl_4_-induced MAPK activation were investigated. MAPKs have been reported to be critical for HSC activation and collagen synthesis. Suppression of ERK activation was associated with complete inhibition of HSC proliferation in vitro [41]. Inhibition of JNK activity in quiescent HSCs or in culture-activated HSCs can prevent HSC activation [42]. During the early period of HSC activation, TGF-β_1_ promotes HSC activation through the p38 MAPK signalling pathway and leads to the formation of hepatic fibrosis [43]. Furthermore, the application of specific inhibitors of ERK, JNK, and p38 could reduce platelet derived growth factor (PDGF)-induced activation of each targeting pathway to completely inhibit HSC activation and collagen expression [44,45]. In this study, the increased levels of phosphorylated JNK, ERK, and p38 were observed after CCl_4_ administration, which were significantly reversed by AG. Therefore, the significant protective effects of AG on CCl_4_-induced hepatic fibrosis in mice are at least partially attributable to decreased JNK, ERK, and p38 phosphorylation through the MAPK cell-signalling pathway.

In conclusion, AG exhibits efficacious anti-fibrotic effects on CCl_4_-induced liver injuries by improving liver function and the histopathological appearance of the liver tissue. The molecular mechanism appears to be anti-oxidative properties and suppressing the MAPK signalling pathway. AG can be obtained widely and cheaply from raw material with few side effects and has efficacious inhibitory effect on hepatic fibrosis. Thus, AG supplementation may be a potential therapeutic strategy for treating liver fibrosis. These findings lay a foundation for improving liver fibrosis and bring us hope for the future control of liver fibrosis and cirrhosis.

## 4. Materials and Methods

### 4.1. Reagents and Chemicals

Amarogentin (C_29_H_30_O_13_, CAS#: 21018-84-8, MW 586.54, HPLC > 99%) was provided by Xi’an Day Natural Inc. (Xi’an, China, no. 20130425). CCl_4_ was purchased from Tianjing Fuyv Chemical Reagent Co. Ltd. (Tianjing, China, no. 20150321), and olive oil was purchased from Chengdu Kelong Chemical Reagent Factory (Chengdu, China, no. 20110510). Colchicine (99%, purity) was purchased from the Yunnan Phytopharmaceutical Co. Ltd. (Kunming, China, no. 20141203). Sodium carboxymethylcellulose (Tianjing, China, No. 20110809) and paraformaldehyde (Tianjing, China, no. 20130924) were obtained from Tianjing Kemiou Chemical Reagent Co. Ltd (Tianjing, China). A MAPK Family Antibody Sampler Kit, Bcl-2, Bax, and GADPH antibodies were purchased from Cell Signaling Technology (Beverly, MA, USA), and the α-SMA and TGF-β_1_ antibodies were purchased from Abcam Inc. (Cambridge, MA, UK). All other reagents, unless indicated, were obtained from Sigma Chemical Co. (Saint Louis, MO, USA).

### 4.2. HSC Culture and CCK-8 Assay

The HSCs were grown in Dulbecco's Modified Eagle medium (DMEM) supplemented with 10% (*v/v*) fetal bovine serum, penicillin (100 U/mL), and streptomycin (100 U/mL), and maintained in a humidified atmosphere of 5% CO_2_ at 37 °C. AG was dissolved in DMEM medium with the concentration diluted at 0.01, 0.1, and 1 mg/mL. Cells were seeded in 96-well plates at a density of 1 × 10^4^ cells per well and incubated overnight. HSC were stimulated with or without TGF-β_1_ (12 ng/mL) for 24 h. Then AG (0.01, 0.1, 1 mg/mL) was added. After 48 h of incubation, 20 μL of CCK-8 was added and incubated for 4 h. The absorbance at 450 nm was recorded using a Model 680 microplate reader (Bio-Rad, Hercules, CA, USA).

### 4.3. Animals and Treatment

Healthy male C57BL/6 mice (18 ± 2 g) were purchased from the Experimental Animal Research Center at the Fourth Military Medical University (Xi’an, China). The animals were maintained in individually-ventilated cages during the experiment and were provided standard laboratory food and water ad libitum. The experimental protocol (2015-0812-R) was reviewed and approved by the Institutional Animal Care and Use Committee of the Fourth Military Medical University.

After one week of acclimation, the C57BL/6 mice were randomly divided into the following six groups: normal group, CCl_4_ group, colchicine group (positive control, 0.1 mg/kg), and AG groups (25, 50, 100 mg/kg). Dose of CCl_4_, colchicine and AG were selected on the basis of previous study [11,46]. Animals in the CCl_4_, colchicine and AG groups were administered 20% CCl_4_ dissolved in olive oil (6 mL/kg) via subcutaneous injection twice per week (Monday and Thursday) for seven consecutive weeks to establish the liver fibrosis mode. The control group was administered 6 mL/kg olive oil at the same time points. After one week of CCl_4_ administration, to prevent invasion of CCl_4_, the control and CCl_4_ groups received equal quantities of normal saline; colchicine (0.1 mg/kg) and AG (25, 50, 100 mg/kg) were suspended in a 0.5% sodium carboxymethylcellulose solution, respectively, and administered by oral gavage every day for six consecutive weeks.

Twelve hours after the last dose of AG, the animals were sacrificed with an overdose of sodium pentobarbital. Blood specimens were collected via retro-orbital bleeding, and serum was subsequently separated by centrifugation at 3500 rpm/min for 10 min and stored at −80 °C until the biochemical assays were performed. The liver tissues were rapidly dissected, washed immediately with ice-cold normal saline and their weights were recorded. The liver samples were either immediately frozen in liquid nitrogen and kept at −80 °C or fixed with 4% paraformaldehyde and embedded in paraffin for histological examination.

### 4.4. Acute Toxicological Test

The mice were orally administered AG (1000 mg/kg) or vehicle. The general behaviours, such as motor activity, tremors, convulsions, aggressiveness, loss of lighting reflex, sedation, muscle relaxation, hypnosis, analgesia, ptosis, lacrimation, diarrhoea, and skin colour were observed for 14 days.

### 4.5. Biochemical Analysis 

Serum levels of ALT, AST, and Alb were used to assess liver function. The GSH-Px, SOD, and MDA levels in liver tissues were used to estimate the level of lipid peroxidation products and antioxidants. Hepatic cGMP level, reflecting hepatic NO bioavailability, was measured by enzyme immunoassay. As a major component of the collagen protein, the Hyp level was used to assess the degree of liver fibrosis. They were all measured with commercial kits (Nanjing Jiancheng Bioengineering Institute, Nanjing, China) using a half-automatic biochemical analyser, according to the manufacturer’s instructions.

### 4.6. Histopathology Examinations

The fixed paraffin tissues were dehydrated in a graded ethanol series and embedded in paraffin wax. Tissues (3 µm) were placed on glass slides, and paraffin was removed with xylene. Then, the tissue sections were subjected to haematoxylin and eosin, Masson’s Trichrome and Sirius Red staining to evaluate the severity of fibrosis. Examinations were performed and photographs were captured with a light microscope (Eclipse 80i, Nikon, Japan). Histopathological evaluations were performed on four sections per slide for all specimens. ImageJ software (Bio-Rad, Hercules, CA, USA) was used to calculate the ratio of the area with positive expression to the total field of Sirius Red staining.

### 4.7. Immunohistochemical Staining for α-SMA

The paraffin sections were deparaffinized in xylene, dehydrated in a graded series of ethanol, and subjected to antigen retrieval in citrate buffer at 100 °C for 1 min. The sections were allowed to cool to room temperature, washed in phosphate buffered saline (PBS), and incubated in 3% hydrogen peroxide for 15 min to block endogenous peroxidase activity, and then in 5% bovine serum albumin (BSA) for 30 min. Next, sections were incubated with a primary antibody against α-SMA (1:100 dilutions in PBS) overnight at 4 °C. Sections were then rinsed (PBS) and incubated with species-specific peroxidase-conjugated secondary antibodies (Boster Biotechnology, Shanghai, China) for 1.5 h. The reaction was visualized with a solution of 3,3′-diaminobenzidine. Subsequently, sections were counterstained with haematoxylin, and a positive signal was detected as a brown colour under a light microscope. At least four randomly-selected fields from each section were examined at 100× magnification and analysed using ImageJ software.

### 4.8. Western Blot Analysis

Cells or liver tissue homogenates were lysed in ice-cold Radio-Immunoprecipitation Assay (RIPA) buffer (50 μM Tris/HCl, pH at 7.4, 150 μM NaCl, 1% NP-40, 0.5% deoxycholate, 0.1% sodium dodecyl sulphate) with a 1% protease and phosphatase inhibitor cocktail and sonicated three times for 10 s per time. The total protein concentrations were measured using a bicinchoninic acid (BCA) Protein Assay kit (Beyotime Biotechnology, Shanghai, China). Samples containing equal amounts of protein were separated by 10% sodium dodecyl sulphate polyacrylamide gel electrophoresis (SDS-PAGE) and transferred onto 0.45-μm polyvinylidene fluoride membranes (Millipore Corp., Bedford, MA, USA). Membranes were blocked with 5% skimmed milk in Tris-buffered saline, pH 7.4, containing 0.1% Tween-20 at room temperature for 2 h and then incubated with the following primary antibodies overnight at 4 °C: α-SMA (1:1000), TGF-β_1_ (1:1000), ERK (1:1000), JNK (1:1000), p38 (1:1000), p-ERK (Thr202/Tyr204) (1:1000), p-JNK (Thr183/Tyr185) (1:1000), p-p38 (Thr180/Tyr182) (1:1000), Bcl-2 (1:1000), and Bax (1:1000). The membrane was simultaneously incubated with an antibody against GADPH (1:1000) to normalize protein loading. After incubation with the primary antibodies, the membranes were washed and incubated with the appropriate horseradish peroxidase-conjugated secondary antibodies for 2 h at room temperature. The transferred proteins were visualized with an enhanced chemiluminescence detection kit (Millipore Corp., Billerica, MA, USA). Bands were imaged by scanning densitometry and quantified using ImageJ software. Band intensity was expressed as a ratio of the protein of interest and GADPH. All experiments were repeated three times.

### 4.9. Statistical Analysis

All reported values are presented as the means ± standard deviation (SD). One-way analysis of variance (ANOVA) with Tukey’s test was used for multiple comparisons in GraphPad Prism 5.0 (GraphPad Software, Inc., La Jolla, CA, USA). *p* < 0.05 was accepted as the level of statistical significance.

## Figures and Tables

**Table 1:** Effects of AG on relative liver weights and liver function in carbon tetrachloride (CCl_4_)-treated mice.

Groups	Control	CCl_4_	Colchicine	AG (25 mg/kg)	AG (50 mg/kg)	AG (100 mg/kg)
Initial body weight (g)	19.26 ± 1.89	20.75 ± 1.33	19.75 ± 1.02	20.83 ± 1.34	20.88 ± 1.86	20.96 ± 1.54
Final body weight (g)	21.74 ± 1.68	22.45 ± 0.81	22.14 ± 1.16	23.19 ± 1.92	23.25 ± 1.20	23.38 ± 0.60
Weight increase (g)	2.48 ± 0.31 ^#^	1.70 ± 0.22 *	2.39 ± 0.25 ^#^	2.36 ± 0.21 ^#^	2.37 ± 0.26 ^#^	2.42 ± 0.30 ^#^
Liver weight (g)	0.93 ± 0.12	1.15 ± 0.15	1.05 ± 0.09	1.12 ± 0.27	1.10 ± 0.11	1.05 ± 0.08
Relative liver weight (%)	4.26 ± 0.46 ^#^	5.13 ± 0.45 *	4.74 ± 0.35	4.82 ± 0.57	4.73 ± 0.26	4.48 ± 0.36 ^#^
ALT (U/L)	19.75 ± 4.74 ^#^	58.53 ± 6.30 *	26.02 ± 6.92 ^#^	38.29 ± 5.89 *^#^	29.56 ± 6.56 ^#^	26.07 ± 4.60 ^#^
AST (U/L)	36.05 ± 8.24 ^#^	124.59 ± 6.48 *	58.81 ± 8.81 *^#^	89.82 ± 6.30 *^#^	74.75 ± 8.26 *^#^	55.86 ± 4.29 ^#^
Alb (g/L)	22.27 ± 3.92 ^#^	11.22 ± 1.65 *	15.47 ± 2.69 *	13.44 ± 1.22 *	14.36 ± 2.77 *	20.90 ± 4.01 ^#^
cGMP (pmol/mg)	1253.95 ± 206.13 ^#^	561.38 ± 92.87 *	854.96 ± 142.36 *^#^	689.69 ± 119.36 *	842.81 ± 135.64 *^#^	965.58 ± 180.36 *^#^

Relative liver weight = liver weight/body weight × 100%. Values are expressed as the means ± standard deviation (SD) (*n* = 7–8) and were analysed using one-way analysis of variance (ANOVA) with Tukey’s multiple comparisons test. * *p* < 0.05 versus the control group; ^#^ *p* < 0.05 versus the CCl_4_ group. ALT: alanine aminotransferase; AST: aspartate aminotransferase; Alb: albumin; cGMP: cyclic guanosine monophosphate.

## Supplementary Materials

The following are available online. Figure S1: Effect of amarogentin on HSC proliferation and apoptosis in vitro, Table S1: Effects of amarogentin on survival rates in CCl_4_-treated mice.
