# Supplementary material for: Protective Effects of Amarogentin against Carbon Tetrachloride-Induced Liver Fibrosis in Mice
Source: Molecules. 2017 May 6;22(5):754. doi: 10.3390/molecules22050754 (PMC6154739; doi:10.3390/molecules22050754)
Supplement: Supplementary file 1 [file molecules-22-00754-s001.pdf]

Supplementary Table S1. Effects of amarogentin on survival rates in CCl_4-treated mice

Groups	No.tested	Survivors	%Survivors
Control	8	8	100
CCl_4	8	7	87.5
Colchicine	8	8	100
AG (25 mg/kg)	8	7	87.5
AG (50 mg/kg)	8	8	100
AG (100 mg/kg)	8	8	100

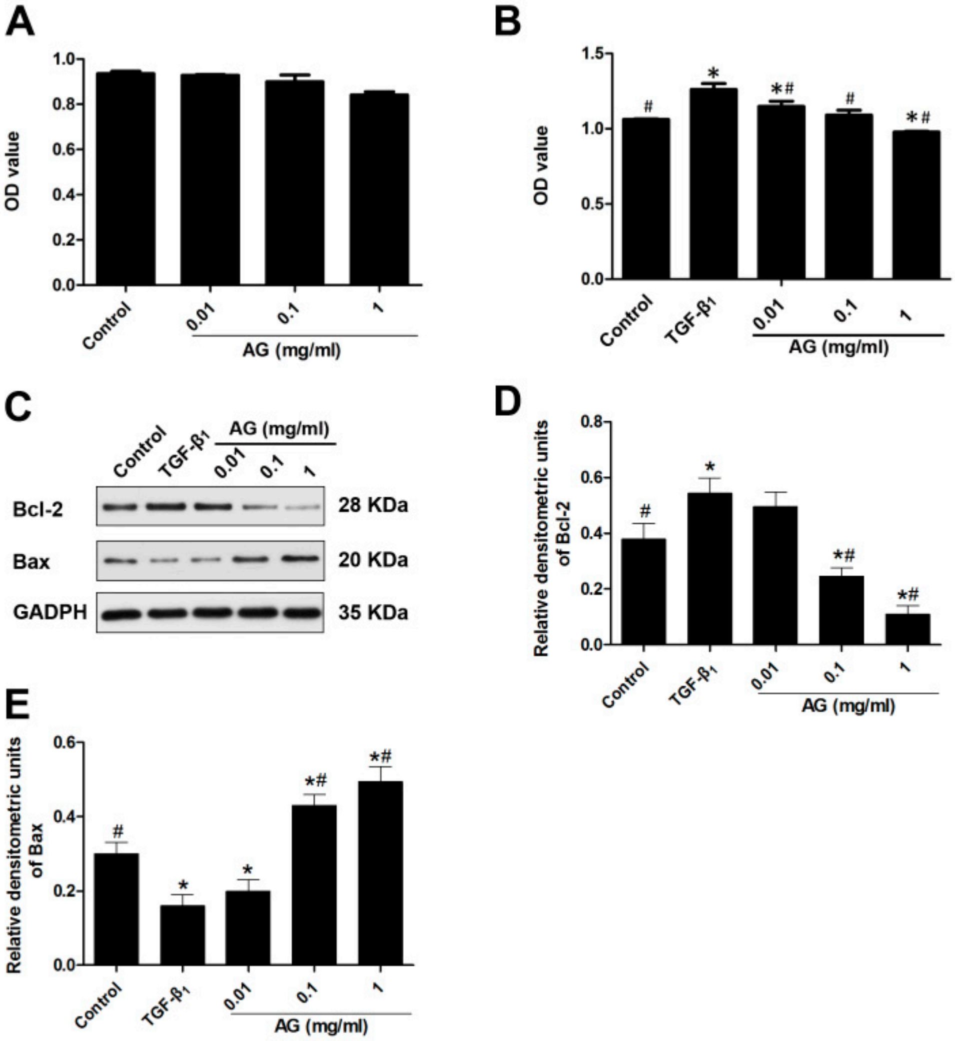

Supplementary Figure S1. Effect of amarogentin on HSC proliferation and apoptosis *in vitro*. (A) Effect of AG on HSC proliferation; (B) effect of AG on TGF-β_1-induced HSC proliferation. (C) immunoblots of the Bcl-2 and Bax protein expression levels in HSC; (D) densitometry analysis of Bcl-2; (E) densitometry analysis of Bax. Values are expressed as the means ± SD (values of three independent experiments) and were analysed using one-way ANOVA with Tukey's multiple comparisons test. * $p < 0.05$ versus the control group, # $p < 0.05$ versus the TGF-β_1 group.
